# CDCA5 functions as a tumor promoter in bladder cancer by dysregulating mitochondria-mediated apoptosis, cell cycle regulation and PI3k/AKT/mTOR pathway activation

**DOI:** 10.7150/jca.35372

**Published:** 2020-02-10

**Authors:** Guanghou Fu, Zhijie Xu, Xiaoyi Chen, Hao Pan, Yiming Wang, Baiye Jin

**Affiliations:** Department of Urology, The First Affiliated Hospital, School of Medicine, Zhejiang University, Hangzhou, Zhejiang 310009, China

**Keywords:** bladder cancer, cell division cycle associated 5, cell cycle, PI3K/AKT/mTOR pathway, apoptosis

## Abstract

Bladder cancer (BC) is one of the most prevalent cancers worldwide and has high rates of relapse and progression. Cell division cycle associated 5 (CDCA5), a substrate of the anaphase-promoting complex, was reported to be upregulated in several types of cancer; however, the function of CDCA5 in BC remains unclear. In this study, we observed that BC tissues had higher levels of CDCA5 expression than adjacent normal tissues. We also found that high CDCA5 expression in patients was associated with poor survival rates. An in vitro study showed that knockdown of CDCA5 in T24 and 5637 cells reduced cell proliferation and induced apoptosis in T24 and 5637 cells, while overexpression of CDCA5 in UMUC3 cells caused the opposite effects. In an additional experiment, we found that CDCA5 promoted cell proliferation by upregulating two key cell cycle factors, cell division cycle protein 2 (CDC2) and cyclin B1, and by activating the PI3K/AKT/mTOR pathway. Furthermore, CDCA5 regulate cancer cell apoptosis through the mitochondrial apoptosis pathway. In conclusion, CDCA5 plays a pivotal role in the proliferation of BC cells. A better understanding of CDCA5 may provide new insights into its role as a therapeutic target for BC.

## Introduction

Bladder cancer (BC) is the most common tumor arising from the urinary system, with approximately 400000 cases diagnosed annually 1. The incidence rate in men to that in women is approximately 3:1. Most newly diagnosed BC patients (70-80%) have nonmuscle-invasive bladder cancer (NMIBC), among which 50-70% recur and 10-20% progress to muscle-invasive bladder cancer (MIBC)^2^-^4^. Hence, in addition to primary detection tools, such as cytology and cystoscopy with biopsies, it is necessary to determine novel biomarkers for the early diagnosis of BC to improve the prognosis of patients.

The phosphoinositide 3-kinase (PI3K)/AKT signaling pathway is involved in numerous cellular processes, including cell proliferation, metabolism, invasion and migration 5-7. When activated by upstream signals, PI3K will trigger the activation of AKT and phosphoinositide-dependent kinase 1 (PDK1), which subsequently causes the phosphorylation of mammalian target of rapamycin (mTOR). As a vital protein that has been highly conserved throughout evolution, mTOR is activated in 70% of cancers 8 and is often correlated with cell proliferation, differentiation and apoptosis.

Cell division cycle associated 5 (CDCA5), also known as sororin, is a key player in sister chromatid cohesion and separation and was first identified as a substrate of the anaphase-promoting complex 9, 10. Moreover, CDCA5 is necessary for the stable blinding of chromatids during S and G2/M phases, and it is also needed to maintain and repair the stability of DNA strands during G2 phases 9, 11-14. In the G1 phase, the CDCA5 protein is degraded through Cdc20 homolog 1 (Cdh1)-activated APC (APC^cdh1^)-dependent ubiquitination. Recent studies have reported that CDCA5 is overexpressed in some tumor tissues, suggesting that it may act as a promoter in tumor progression 15-20. Although high CDCA5 expression is associated with advanced stages in various tumors, the role of CDCA5 in BC development and progression is not fully defined.

In the current study, we found that BC cells had significantly higher CDCA5 expression than normal bladder cells, and this expression was related to advanced clinical stage and poor prognosis. In addition, knocking down or overexpressing CDCA5 in BC cells affected their proliferation and apoptosis. Taken together, our results showed that CDCA5 may act as a potential therapeutic target in BC.

## Materials and methods

***Tissue samples and immunohistochemistry.***Twenty BC patients sourced from the First Affiliated Hospital of Zhejiang University School of Medicine were enrolled in the study from July 2017 to December 2018. All of these patients had histologically confirmed muscle-invasive urothelial cancer and no metastatic disease present. Patients with metastatic disease who have received prior radiotherapy for BC may be excluded. All participants signed informed consent. Immunohistochemical staining was performed according to the kit instructions. In brief, after paraffin section dewaxing, the sections were rehydrated and subjected to high-pressure antigen retrieval. Next, 3% H_2_O_2_ was used to eliminate the influence caused by endogenous peroxidase activity, followed by blocking with normal goat serum. The sections were incubated with primary antibodies against CDCA5 (1:100; cat no. ab192237; Abcam), Ki67 (1:200; cat no. ab16667; Abcam), cleaved caspase-3 (1:100; cat no. ab2302; Abcam), cyclin B1 (1:200; cat no. ab181593; Abcam) and p-Akt (1:200; cat no. ab81283; Abcam) overnight at 4°C. Following incubation with the secondary antibody (goat anti-rabbit IgG antibody-HRP), DAB was used to stain the slides, and hematoxylin counterstaining and alcohol dehydration were performed. Finally, the prepared sections were examined under a microscope.

***Online database.***The Oncomine database (http://www.oncomine.org) was used to identify CDCA5 mRNA expression in BC and normal tissues with the following search terms: CDCA5 and BC^21^. We analyzed the results of the Lee et al 22 bladder samples in the Oncomine database. Clinical significance of CDCA5 was analyzed by The Cancer Genome Atlas (TCGA) database (https://cancergenome.nih.gov/) using the following search terms:

project, TCGA-bladder urothelial carcinoma; primary site, bladder; access date, January 2018.

***Cell culture.***The human BC cell lines RT4, UM-UC-3, 5637, T24, TCCSUP and the normal uroepithelial cell line SV-HUC-1 were purchased from the Cell Bank of the Chinese Academy of Sciences (Shanghai, China). RT4, 5637, T24 and TCCSUP cells were cultured in RPMI 1640 medium containing 10% fetal bovine serum (FBS, Thermo Fisher Scientific, Gibco); UM-UC-3 cells were cultured in Dulbecco's Modified Eagle's Medium (DMEM) containing 10% FBS, and SV-HUC-1 cells were cultured in Minimum Essential medium (MEM) containing 10% FBS. All of the above cell lines were preserved at 37°C in 5% CO_2_ in a stable incubator.

***Cell transfection.***shRNA vectors for (sh-CDCA5-1,2) and a control vector were purchased from Guangzhou GeneCopoeia Co., Ltd. (Guangzhou, China). The sequences of shRNAs were as follows: sh-CDCA5-1 forward, 5'CCGGGGACGCCAGAGACTTGGAAATCTCGAGATTTCCAAGTCTCTGGCGTCCTTTTTG3' and reverse, 5'AATTCAAAAAGGACGCCAGAGACTTGGAAATCTCGAGATTTCCAAGTCTCTGGCGTCC3'; CDCA5-2 forward, 5'CCGGGACATGACTCTCCCTGGAATCCTCGAGGATTCCAGGGAGAGTCATGTCTTTTTG3' and reverse, 5'AATTCAAAAAGACATGACTCTCCCTGGAATCCTCGAGGATTCCAGGGAGAGTCATGTC3'. The adenoviruses used to express CDCA5 or the negative control (NC) were purchased from GenePharma (Shanghai, China). Before the infection step, the cells were cultured in 6-well plates (5×10^4^ cells/well) to 60% confluence. Then, T24 and 5637 cells were treated with lentivirus at a multiplicity of infection of 50 pfu/cell in 1640 medium with 10% FBS and polybrene (5 μl/ml; Sigma-Aldrich; Merck KGaA); UMUC3 cells were subjected to the same treatment in DMEM. After 48 hours, virally transduced cells were subjected to 1 mg/ml puromycin (Thermo Fisher Scientific) for screening successfully infected cells. Both GFP levels and Western blotting were conducted to validate the transfection efficiency.

***Cell proliferation assay and colony formation assay.***The proliferation rates of cells were investigated using the Cell Counting Kit-8 (Dojindo Molecular Technologies, Inc., Kumamoto Japan) according to the manufacturer′s protocol. Cells were cultured in 96-well plates at a density of 1000 cells per well at 37°C in 5% CO_2_. Then, the cells were treated with 10% CCK-8 reagent for another 1 h at 37°C before the absorbance was measured at 450 nm. The colony formation assay was performed as follows: cells were prepared in 6-well plates at a density of 500 cells per well. After discarding the supernatants after 8-10 days, the cells were washed 3 times with phosphate-buffered saline (PBS), followed by 15 min fixation at room temperature; finally, the cells were stained with 0.5% crystal for 15 min, and the 6-well plate was photographed and analyzed.

***Quantitative real-time PCR.***Total RNA was isolated by TRIzol (Invitrogen; Thermo Fisher Scientific, Inc.) from the BCa cell lines or frozen tissue samples. cDNA was synthesized from 2 µg total RNA using TaKaRa PrimeScript^TM^ RT and SYBR EX Taq^TM^ kits (TaKaRa Bio, Inc., Otsu, Japan), and qRT-PCR was conducted using a Bio-Rad CFX96 real-time system according to the manufacturer's instructions. The primers were designed as follows: for CDCA5, forward primer, 5'CGCCAGAGACTTGGAAATGT3' and reverse primer, 5'GTTTCTGTTTCTCGGGTGGT3'; for β-actin, forward primer, 5'GCAAGCAGGAGTATGACGAG3' and reverse primer, 5'CAAATAAAGCCATGCCAATC3'. Control groups were used to confirm the absence of the pollution of agents or primer dimers, and melt-curve analysis was used to identify the specificity of amplification. All genes were normalized to β-actin expression, and the gene mRNA relative expression levels were calculated using the ΔΔCq method (18).

***Western blotting.***T24, 5637 and UMUC3 cell lines were harvested and washed twice with PBS and lysed using radio-immunoprecipitation assay lysis buffer (Cell Signaling Technology, Danvers, MA, USA) containing 1% of a protease inhibitor cocktail (Thermo Fisher Scientific, Inc.) for 1 hour at 4°C and collecting the supernatants after centrifugation (4°C, 15,000 x g, 15 min). Protein concentrations were measured using a bicinchoninic acid protein assay (Pierce; Thermo Fisher Scientific, Inc.). The samples were boiled at 95°C for 5 min after mixing with 4x sodium dodecyl sulfate (SDS) loading buffer (Invitrogen). The proteins (20 μg/10 μl) were loaded in 12% Tris-acetate gels (Invitrogen; Thermo Fisher Scientific, Inc.) and then separated by electrophoresis on SDS-PAGE gels; the proteins were subsequently transferred onto a polyvinylidene fluoride membrane. The membranes were blocked with 5% nonfat milk for 1 hour at room temperature, and the membranes were blocked and incubated with the corresponding primary antibodies overnight at 4°C. Immunoblots were washed with Tris-buffered saline containing 1% Tween-20 (TBST) three times and incubated with conjugated anti-mouse or anti-rabbit antibodies (1:5000) for 1 hour at room temperature. The primary antibodies used in this experiment were: rabbit polyclonal anti-CDCA5 antibody (1:1,000; cat no. ab192237; Abcam); rabbit monoclonal anti-phosphorylated-protein kinase B antibodies (AKT; Ser473; 1:1,000; cat no. ab81283; Abcam), anti-AKT (1:1,000; cat no. #4685; CST), anti-P21 (1:2,000; cat no. ab109520; Abcam), anti-P27 (1:2,000; cat no. ab32034; Abcam), anti-cyclin B1 (1:1,000; cat no. ab181593; Abcam), anti-Chk2 (1:1,000; cat no. ab109413; Abcam), anti-Parp (1:1,000; cat no. #9532S; CST), anti-Caspase-9 (1:1,000; cat no. ab202068; Abcam), anti-Caspase-3 (1:1,000; cat no. ab32351; Abcam), anti-Bcl-xL (1:1,000; cat no. ab32370; Abcam), anti-Bcl2 (1:1,000; cat no. ab32124), anti-Bax (1:1,000; cat no. ab32503; Abcam), anti-PI3k (1:2,000; cat no. #3011S; CST), anti-phospho-PI3 kinase (p-PI3K; 1:1,000; cat no. #4228; CST), anti-mTOR (1:1,000; cat no. #2983; CST), anti-phospho-mTOR (p-mTOR; Ser2448; 1:1,000; cat no. #5536; CST), mouse monoclonal anti-β-actin antibody (1:2,000; cat no. ab6276; Abcam), and anti-CDC2 (CDK1; 1:1000; cat no. ab18; Abcam). Autoradiography with the ChemiDoc MP Imaging System (Bio-Rad) with an EZ-ECL Chemiluminescence Detection Kit (Biological Industries, Kibbutz Beit Haemek, Israel), and β-actin was selected as an internal reference.

***Flow cytometry analysis of cell apoptosis.***Stably transfected T24, 5637 and UMUC3 cells were seeded in a 6-well plate at a density of 2x10^5^ cells/well. Cells were harvested by non-EDTA trypsin (Biological Industries), washed twice with PBS and subsequently incubated using a BD Annexin V-APC/7-aminoactinomycin D Apoptosis Detection Kit (BD Biosciences, San Jose CA, USA). Finally, the apoptosis cells were measured by staining with Annexin V (10 μl/200 μl) along with 7-aminoactinomycin D (10 μl/200 μl) After incubating for 15 min at room temperature, the stained cells were detected using BD FACSCanto^TM^ II (BD Biosciences), and apoptotic cells were analyzed using FlowJo 7.6 software (FlowJo LLC, Ashland, OR, USA).

***Flow cytometry analysis of the cell cycle.***Stably transfected T24, 5637 and UMUC3 cells were seeded in 6 cm dishes at a density of 60%. A total of 1.0×10^6^ transfected cells were collected in PBS, fixed in 70% ethanol overnight at -20°C, subsequently washed twice with PBS and harvested by centrifugation at 1000 rpm for 5 min. Cells were resuspended in a cell cycle staining kit (Multisciences, Hangzhou, People's Republic of China) according to the manufacturer's protocol and incubated for 30 min in the dark at room temperature. All cells were measured using BD FACSCanto^TM^ II (BD Biosciences), and the results were analyzed using FlowJo 7.6 software (FlowJo LLC, Ashland, OR, USA).

***Experimental animals and tumor growth in nude mice.***Ten four-week-old female NOD-SCID mice were purchased from Vital Laboratory Animal Technology Co., Ltd. (Beijing, China). They were raised in the animal facility of Zhejiang University School of Medicine. All experimental procedures and protocols were performed in compliance with the Animal Experimental Ethical Committee of the First Affiliated Hospital, College of Medicine, Zhejiang University and were approved by the Zhejiang Medical Experimental Animal Care Commission. To establish the subcutaneous model, lentivirus-transfected T24 cells (5×10^6^ cell/ml) with a low expression of CDCA5 and the NC cells were suspended in 0.2 mL of PBS, which was subcutaneously injected into the left flank of each mouse. We observed the survival of these mice and the growth of the tumors. The mice were monitored once every 2 days. All nude mice were sacrificed by cervical dislocation 7 weeks later; the tumors were removed; and the relative data, such as the length, width and weight, of the tumor were recorded.

***Statistical analysis.***SPSS 22.0 (IBM Corp.) was used to analyze the data. The normality of the data was initially determined using a Kolmogorov-Smirnov test. Data are shown as the mean ± standard deviation. The correlations between CDCA5 expression and clinicopathological characteristics were analyzed using a Pearson's χ2 test or a continuity correction χ2 test. The survival rate was evaluated using the Kaplan-Meier method, and data were analyzed by a log-rank test. The significant differences between two groups were determined using a two-tailed Student's t-test. Student-Newman-Keuls test was used as a post hoc test. P<0.05 was recognized as statistically significant. Data were obtained from triplicate and three or more independent experiments.

## Results

**Figure 1:**
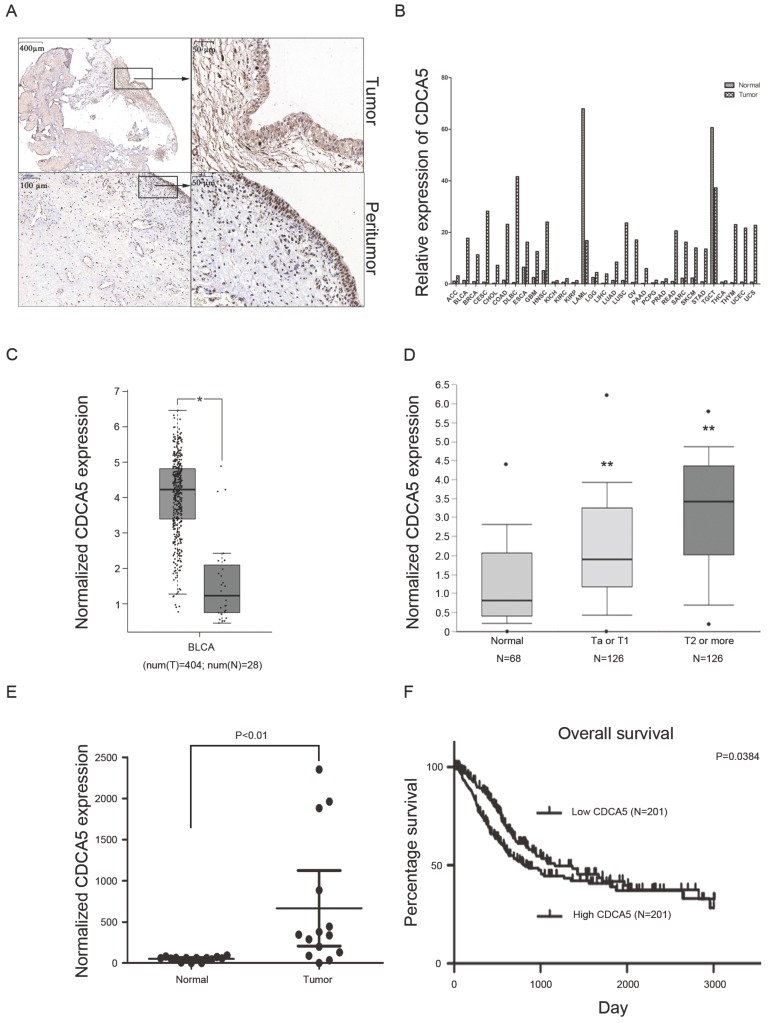
High CDCA5 expression is a poor prognostic indicator in BC. (A) Immunohistochemistry staining of CDCA5 in BC tissue and peritumor tissue. Magnification: 40× and 200×. (B) Relative CDCA5 expression in various types of cancer. The data were based on the TCGA database. (C) CDCA5 expression in BC tissues (N=404) and normal bladder tissues (N=28) from the GEPIA website. (D) Normalized CDCA5 mRNA expression in BC tissues and normal bladder tissues from the Oncomine database (N=320). **P<0.01 Ta or T1 vs. normal and **P<0.01 T2 or more vs. normal. (E) Normalized CDCA5 mRNA expression in 20 pairs of BC tissues; BC tissues vs. normal bladder tissues. (F) OS rates stratified by low CDCA5 expression (N=201) and high CDCA5 expression from the TCGA database. The median was used as the dividing line for high or low CDCA5 expression. BC patients with high expression of CDCA5 had a worse OS rate than those with low expression of CDCA5. CDCA5, cell division cycle associated 5.

***CDCA5 is upregulated in BCa tissues, and high CDCA5 expression indicates poor survival.***Recently, a study revealed that overexpression of CDCA5 was an indicator of poor prognosis in BCa 16. Our results also showed that compared with matched peritumor tissues, bladder tumor tissues highly expressed CDCA5 (Fig. 1A). Using the GEPIA database, the present study also revealed that CDCA5 expression was significantly higher in tumor tissues than in matched normal tissues among various tumors (Fig. 1B). For BCa, CDCA5 expression was higher in bladder cancer tissues than in matched adjacent normal tissues (P<0.05; Fig. 1C). In addition, data from the Oncomine platform showed that CDCA5 was dramatically increased in the Lee et al bladder samples (Fig. 1D; NMIBC vs. normal: fold change, 1.939; t-test, 5.493; P=7.53E-8<0.05; NIBC vs. normal: fold change, 3.751; t-test, 8.217; P=2.42E-13<0.05). In addition, we performed qPCR analysis using cDNA from 20 paired BCa and matched normal bladder tissue samples and found an increased CDCA5 mRNA expression level in the BCa samples (P<0.05; Fig. 1E). To determine the relationship between CDCA5 expression and the survival rates of patients with BCa, we investigated 408 BCa patients from the TCGA database. The results showed a significant association between high and low CDCA5 expression in grade (P<0.001) and metastasis (P<0.05) (Table 1). For the Kaplan-Meier survival analysis, 402 patients were available (as the data for 6 patients was inaccessible), and high CDCA5 expression was related to worse overall survival rates (P=0.0384) (Fig. 1F), indicating that high CDCA5 expression might be a poor prognostic factor in BCa.

**Figure 2:**
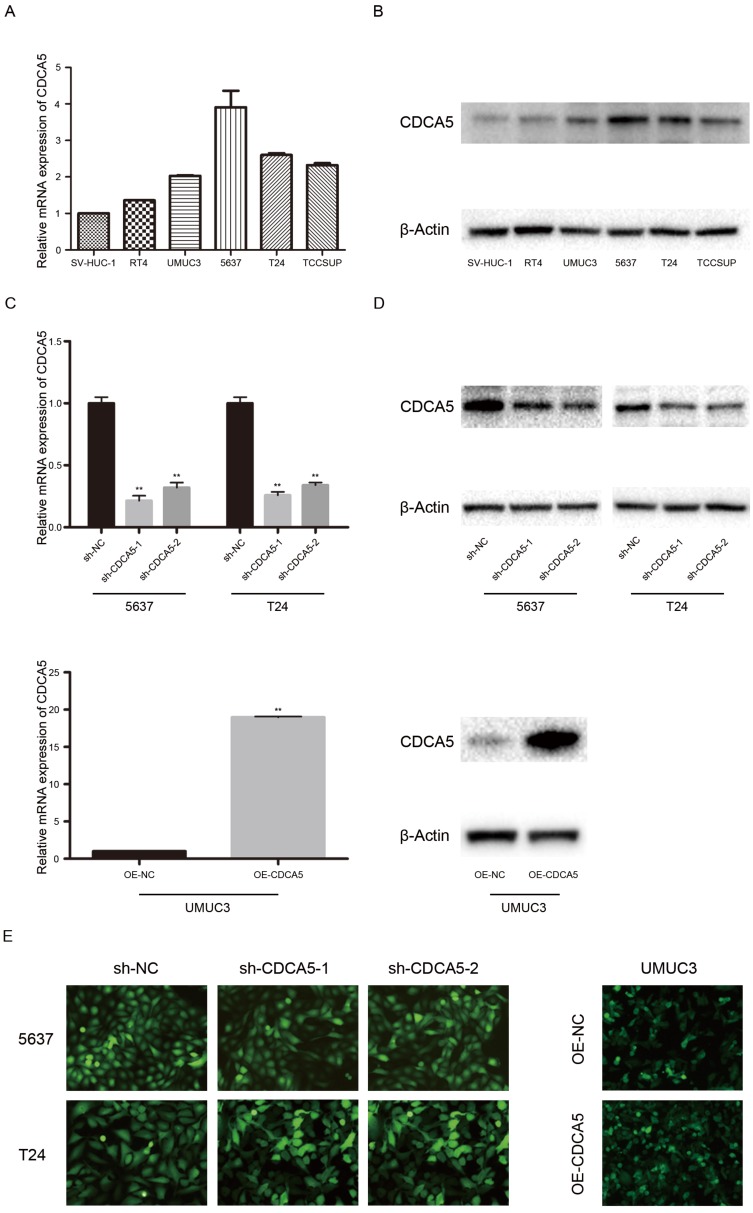
Expression of CDCA5 in BC cell lines. (A) Relative mRNA expression of CDCA5 in BC cell lines (RT4, UMUC3, 5637, T24 and TCCSUP) and a normal bladder uroepithelial cell line (SV-HUC-1). (B) Protein expression of CDCA5 in BC cell lines (RT4, UMUC3, 5637, T24 and TCCSUP) and a normal bladder uroepithelial cell line (SV-HUC-1). (C), (D), (E) Transfection efficiency of sh-NC, sh-CDCA5-1, sh-CDCA5-2, OE-NC and OE-CDCA5 was validated by qRT-PCR, Western blotting and fluorescence microscopy. **P<0.01 vs. sh-NC or OE-NC.

***Expression of CDCA5 in BC cell lines.*** CDCA5 expression was high in the BC cell lines RT4, UMUC3, 5637, T24, and TCCSUP and low in the normal uroepithelial cell line SV-HUC-1 (Fig. 2A, B). In addition, T24 and 5637 cells, which show a higher malignant potential than RT4 cells, expressed higher CDCA5 levels than RT4 cells. To perform further studies, we established T24- and 5637-CDCA5-knockdown models using sh-CDCA5 lentivirus. The infection efficiency was determined using the combination of qPCR analysis, Western blotting and fluorescence microscopy (Fig. 2C, D, E). Additionally, UMUC3, which exhibited lower CDCA5 expression, was transfected with the OE-CDCA5 lentivirus, and its infection efficiency was also confirmed (Fig. 2C, D, E).

**Figure 3:**
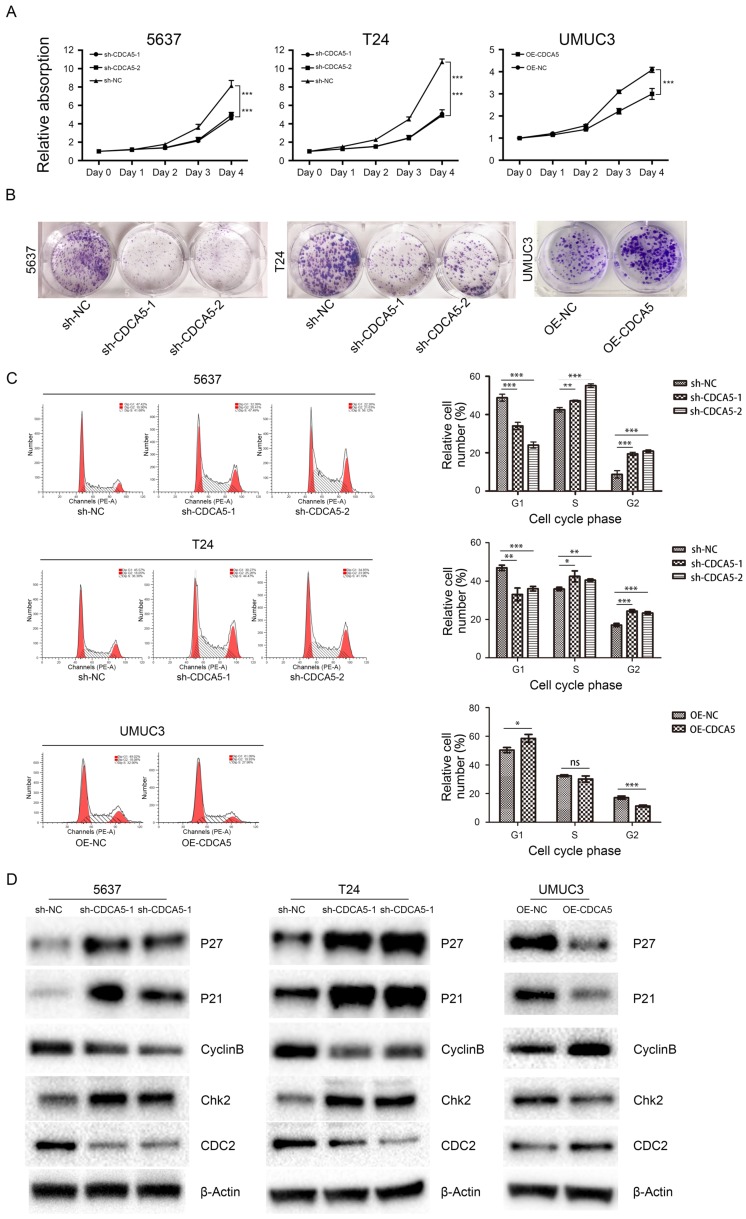
Effect of CDCA5 on the proliferation and regulation of BC cells. (A) Knockdown of CDCA5 inhibited the proliferation of 5637 and T24 cell lines, while overexpression of CDCA5 promoted the proliferation of the UMUC3 cell line as determined by a cell counting kit-8 assay. (B) Colony formation assays were used to detect the proliferation ability of CDCA5 knockdown or overexpression BC cell lines. (C) Flow cytometry was used to calculate the cell cycle distribution following treatment with sh-NC, sh-CDCA5-1, and sh-CDCA5-2 in the 5637 and T24 cell lines and OE-NC and OE-CDCA5 in the UMUC3 cell line. *P<0.05 vs. sh-NC or OE-NC, **P<0.01 vs. sh-NC or OE-NC, ***P<0.005 vs. sh-NC or OE-NC. ns, not significant. (D) Effect of CDCA5 knockdown and overexpression on the expression of cell cycle-related proteins by Western blotting.

***CDCA5 is essential for BCa cell proliferation and cell cycle.***The Cell Counting Kit-8 assay showed that knockdown of CDCA5 in 5637 and T24 cell lines significantly hindered BCa cell proliferation (P<0.05; Fig. 3A), while overexpression of CDCA5 in UMUC3 cells promoted cell proliferation (P<0.05; Fig. 3A). Furthermore, colony formation assays revealed that the numbers of colonies and cell survival rates were decreased after CDCA5 knockdown in the two cell lines and were increased after CDCA5 overexpression in UMUC3 cells (Fig. 3B). As CDCA5 significantly affects BCa cell proliferation, we hypothesized that CDCA5 may play an important role in regulating the cell cycle. Our results showed that compared with the control cells, 5637 and T24 cells with knockdown of CDCA5 had a significantly affected cell cycle, in which more cells transited to G2/M phase and fewer G0/G1 phase cells were left, which indicated that knockdown of CDCA5 induced G2/M arrest and cell growth suppression (Fig. 3C). Overexpression of CDCA5 significantly promotes the G2/M transition. Then, we measured the key protein controlling the cell cycle in cells after knockdown or overexpression of CDCA5 (Fig. 3D). Knockdown of CDCA5 upregulated the expression levels of P27, P21 and Chk2 in T24 and 5637 cells. The expression of Cyclin B1 and CDC2 proteins, which are essential for the transitions of G2/M, was decreased after the knockdown of CDCA5 22-25. In contrast, overexpression of CDCA5 in UMUC3 cells showed the opposite results. Consistent with these results, knockdown of CDCA5 led to fewer G0/G1 phase cells and more G2/M phase cells. As a result, G2/M arrest inhibited cell proliferation. Overexpression of CDCA5 showed the opposite effect.

**Figure 4:**
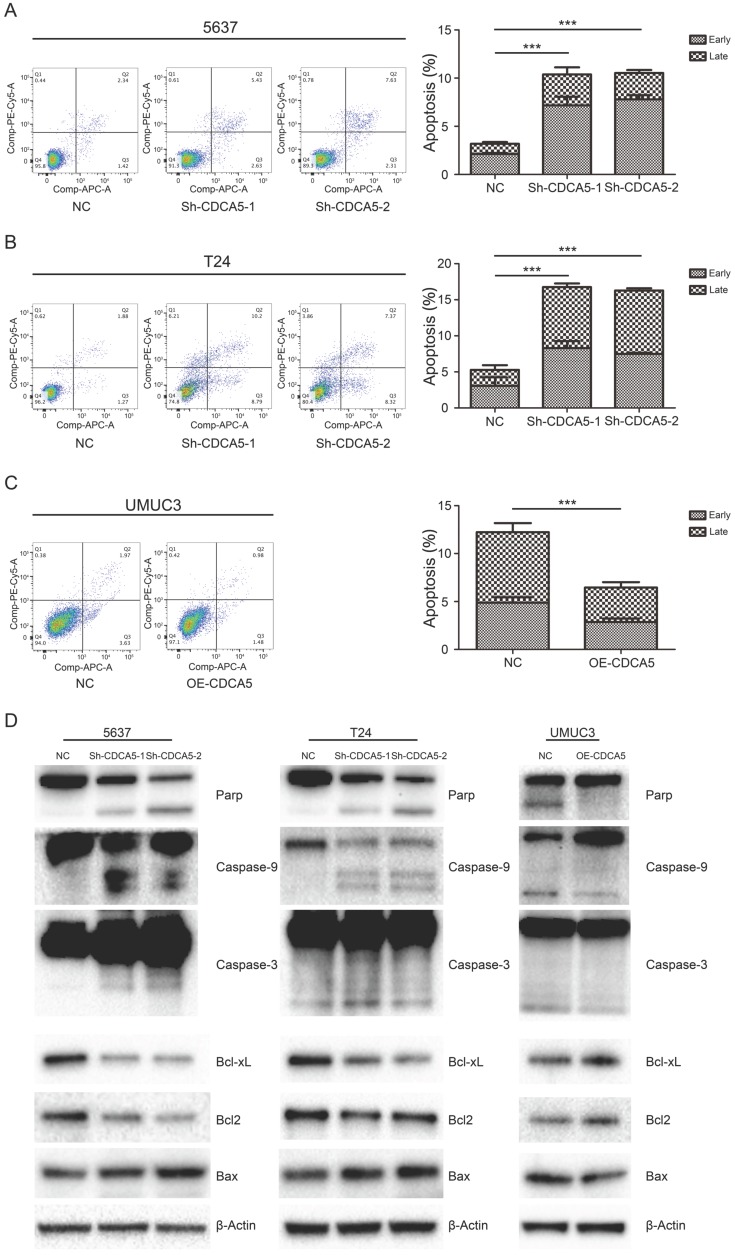
CDCA5 inhibited the apoptosis of BC cells. (A), (B), (C) Flow cytometry analysis was used to present the percentage of apoptosis distribution following treatment with sh-NC, sh-CDCA5-1, and sh-CDCA5-2 in the 5637 and T24 cell lines and OE-NC and OE-CDCA5 in the UMUC3 cell line. ***P<0.005 vs. sh-NC or OE-NC. (D) Effect of CDCA5 knockdown and overexpression on the expression of apoptosis-related proteins by Western blotting.

***CDCA5 inhibited mitochondrial-mediated apoptosis in BCa cells.***We assessed the effect of CDCA5 on the apoptosis of BCa cells using Annexin V and 7AAD staining. Knockdown of CDCA5 significantly promoted apoptosis in the CDCA5-knockdown groups compared with that in the control groups (P<0.05; Fig. 4A, B), whereas CDCA5 overexpression inhibited apoptosis in UMUC3 cells (P<0.05; Fig. 4C). For T24 cells, a 3.2-fold increase in the percentage of apoptotic cells (early apoptotic and late apoptotic cells) was observed after knockdown of CDCA5. The total number of apoptotic 5637 cells increased by 3.3-fold compared to the total number of apoptotic control cells following knockdown of CDCA5. For UMUC3 cells, the number of apoptotic cells dropped from 47.1% of the control and from 4.89% to 2.59% after knockdown. The mean percentage of apoptotic cells treated with sh-NC, sh-CDCA5-1 and sh-CDCA5-2 were 5.27±3.15%, 16.75±2.27% and 16.28±0.76% in T24 cells, and 3.19±0.50% (Sh-NC), 10.38±2.56% (Sh-CDCA5-1) and 10.54±1.25% (Sh-CDCA5-2) in the 5637 cell line, respectively. Moreover, the number of apoptotic cells from the UMUC3 cell line treated with sh-NC and OE-CDCA5 were 4.90±0.90% and 2.59±0.61%, respectively. To study whether CDCA5 affected BCa cell apoptosis through mitochondria-mediated signaling, we measured the expression of intrinsic pathway-related proteins by Western blotting. Knockdown of CDCA5 significantly inhibited the expression of the antiapoptotic proteins BCL-2 and Bcl-x and induced the expression of the proapoptotic protein Bax (Fig. 4D). The expression of downstream proteins of mitochondrial apoptosis, such as cleaved caspase-3, caspase-9, and cleaved PARP, was also significantly elevated in CDCA5-knockdown cells (Fig. 4D). Furthermore, CDCA5 overexpression played an opposite role in the expression of apoptosis-related proteins (Fig. 4D). These findings suggest that CDCA5 is essential in the process of mitochondria-mediated apoptosis.

**Figure 5:**
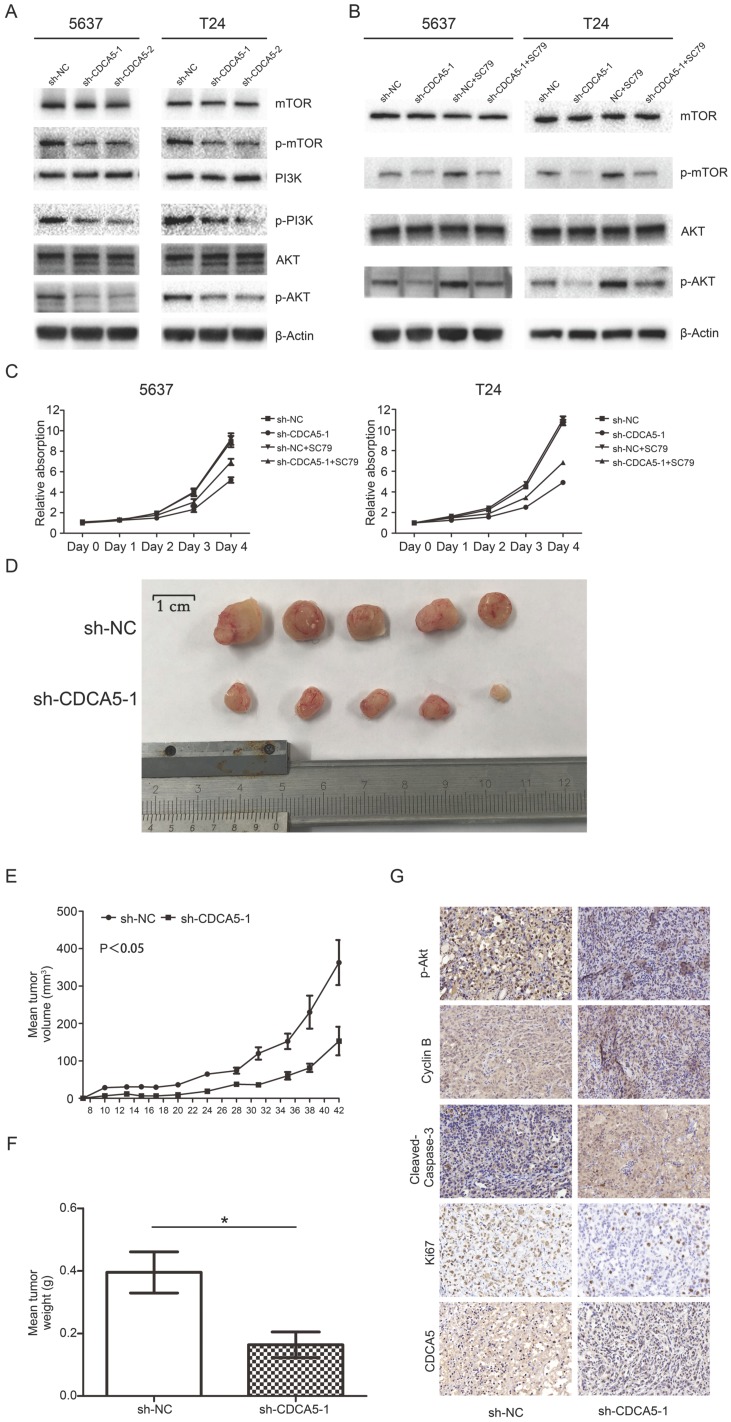
Knockdown of CDCA5 suppressed the PI3k/AKT/mTOR pathway, inhibited tumor growth and induced apoptosis of BC cells in vivo. (A) The levels of the main proteins on the PI3k/AKT/mTOR pathway (mTOR, p-mTOR, PI3K, p-PI3K, AKT, and p-AKT). A rescue assay using 5 µg/ml SC79 partially restored the inhibition of the PI3k/AKT/mTOR pathway due to the sh-CDCA5-1-mediated knockdown of CDCA5. Changes in the protein levels of mTOR, p-mTOR, AKT and p-AKT (B) and the proliferation rates of sh-NC, sh-CDCA5, sh-NC+SC79 and sh-CDCA5+SC79 in 5637 and T24 cell lines, as determined by a cell counting kit-8 assay (C). (D) Harvested tumors from the sh-NC group and sh-CDCA5 group (N=5). (E) Mean tumor volume of the sh-NC group and sh-CDCA5 group. (F) Mean tumor weight of the two groups. (G) Expression of p-AKT, cyclin B1, cleaved caspase-3, Ki67 and CDCA5 in tumors removed from nude mice of the two groups. Magnification 200×. P-values obtained by using one-way analysis of variance.

***Knockdown of CDCA5 regulated cell proliferation by suppressing the PI3K/AKT/mTOR signaling pathway.***To further investigate the mechanism by which CDCA5 affects BCa cell proliferation, we conducted Western blotting to measure the expression of related molecules. Multiple studies have revealed that the PI3K/AKT pathway plays a vital role in the proliferation and apoptosis of various tumors, which also contribute to promoting the progression of BC 26. Thus, the present study investigated whether CDCA5 affected BCa cell proliferation through the PI3K/Akt pathway. As shown in Fig. 5A, compared with scramble control cells, the expression of phosphorylated PI3K (p-PI3K), p-AKT and p-mTOR was inhibited in sh-CDCA5-1 and sh-CDCA5-2 cells, and there were no significant changes in total AKT, total PI3K and total mTOR. Subsequently, we used a specific AKT activator, SC79, to verify the role of the PI3K/AKT/mTOR signaling pathway in CDCA5 knockdown-induced apoptosis. As shown in Fig. 5B, SC79 (5 μg/mL) markedly activated AKT phosphorylation and mTOR phosphorylation in CDCA5-knockdown cells but did not significantly affect the total AKT or total mTOR (Fig. 5B). Furthermore, SC79 could rescue the partial effects of CDCA5 knockdown (Fig. 5C). These results suggest that the oncogenic effects of CDCA5 are associated with the PI3K/AKT/mTOR signaling pathway.

***Knockdown of CDCA5 inhibits the tumor growth of BCa cells in vivo.***To further confirm the effect of CDCA5 on BC growth in vivo, we injected sh-NC and sh-CDCA5-1 T24 cells subcutaneously into the flanks of NOD-SCID mice to establish a xenograft tumor model. All of the sh-CDCA5-1 (n=5) and NC (n=5) groups developed xenograft tumors in the right flank, and xenograft tumors were harvested 6 weeks after injection (Fig. 5D). The NC group developed a larger tumor mass than the sh-CDCA5-1 group according to the average tumor volume and weight (Fig. 5D-F). Furthermore, to explore the mechanism of CDCA5 on BC in vivo, we performed IHC analysis on the tumor mass. The NC group showed more positive staining for Ki67 and cyclin B1 and less positive staining for cleaved caspase-3, indicating that CDCA5 promotes proliferation, regulates the cell cycle, and suppresses the apoptosis of BCa cells. However, the Sh-CDCA5-1 group expressed decreased levels of p-AKT, which showed that p-Akt played a pivotal role in the oncogenic effect of CDCA5 in BC (Fig. 5G).

## Discussion

Tumors are characterized by normal cell growth and the potential to metastasize to other parts of the body. G0 phase cells will turn into the cell cycle when these cells received growth-promoting stimuli, including gene mutations or amplification. The cell cycle of eukaryotic cells is regulated by several molecules, including cyclins; cyclin-dependent kinases (CDKs); CDK-interacting protein/kinase inhibitory protein (cip/kip) family members, such as p21 and p27; and inhibitor of kinase 4/alternative reading frame (INK4a/ARF) family members. The cell cycle contains G1/S, S and G2/M checkpoints 27. The G1/S checkpoint is regarded as the restriction point because of its effect on ensuring sufficient materials for DNA replication and monitoring DNA damage 28. The G2/M checkpoint guarantees the completion of DNA replication, and sister chromatids separate correctly 29. The CDCA5 protein, also called sororin, is encoded by the CDCA5 gene. CDCA5 was first identified as a substrate of the anaphase-promoting (APC) complex and played a role in regulating sister chromatid cohesion 9, 11. CDCA5 also maintains cohesion in chromatin to ensure its maturity and keeps sister chromatids together during mitosis 10. Multiple studies have revealed that CDCA5 is overexpressed in various cancers, such as lung cancer, oral squamous cell carcinoma, gastric cancer, pleural mesothelioma and hepatocellular carcinoma, and acts as a tumor promoter 15, 17-20. Although there was a study that showed that CDCA5 overexpression is associated with advanced clinical features and poor prognosis of urothelial carcinoma, the underlying mechanisms of how CDCA5 promotes the tumorigenicity of BC remain unclear. In the current study, we focused on the impact of CDCA5 in BCa cells and explored the underlying mechanism. Our results first showed that compared with that in matched peritumor tissues, the expression of CDCA5 in BCa tissues was significantly higher. High CDCA5 expression in BCa indicated advanced clinical stage and poor prognosis. Data from the TCGA database showed that patients with MIBC and high TNM stage had high expression levels of CDCA5. The overall survival (OS) and disease-free survival (DFS) rates were worse in patients with a high expression of CDCA5.

Further, we investigated the oncogenic function of CDCA5 in human BCa. Our results showed that CDCA5 knockdown inhibited cell proliferation, promoted cell apoptosis and induced G2/M arrest. With the knockdown of CDCA5 in BCa cells, the expression of cell cycle-related proteins (cyclin B1, CDC2) was downregulated, while p21, p27 and p-Chk2 were upregulated. CDC2 may accelerate the cell cycle G2/M transition when binding to cyclin B1 25, 30-32. Thus, we hypothesized that the G2/M phase arrest induced by CDCA5 knockdown is most likely mediated by CDC2 and cyclin B1. Further, the expression of CDC2 and cyclin B1 was significantly downregulated in CDCA5 knockdown cells. p-Chk2 and p21 are key regulators of the ATM-Chk2/p21-cdc25c signaling pathway, and this pathway plays an important role in regulating the cell cycle 33. p21, which is recognized as a key member of cyclin kinase inhibitors, is closely related to G2/M cell cycle arrest 34. Our results showed that the expression of p21 and p-Chk2 were upregulated after CDCA5 knockdown, which prevented the transition from G2 to M phase. Taken together, we showed that knockdown of CDCA5 induced G2/M arrest, which was mediated by downregulation of cyclin B1 and CDC2 and the activation of the Chk2/p21 pathway.

Apoptosis, also called programmed cell death, can be divided into extrinsic and intrinsic (also known as the mitochondrial pathway) pathways 35. The extrinsic pathway is activated by extracellular surroundings and ligands, which finally induce the formation of the death-inducing signaling complex (DISC). The key proteins in the extrinsic pathway include cell surface receptors, such as the Fas receptor (also known as Apo1 or CD95) and tumor necrosis factor (TNF) 36. The intrinsic apoptosis pathway is usually inhibited in tumor cells 37 and is initiated by the release of proteins from the intermembrane space of mitochondria, especially the Bcl-2/Bax system 38. The Bcl-2/Bax system includes antiapoptotic proteins (Bcl-2 and Bcl-xL) and proapoptotic proteins (Bax, Bak, Bad, Bid, and Bik). The Bax/Bcl-2 ratio is closely related to cell apoptosis; overexpressed Bax promotes apoptosis, while Bcl-2 inhibits apoptosis. Caspase-9 and caspase-3 are downstream executioners in mitochondrial apoptosis. Furthermore, poly (ADP-ribose) polymerase (PARP) can be activated when cells experience stress and/or DNA damage to process apoptosis. In our study, CDCA5 knockdown significantly promoted apoptosis, and the expression of cleaved caspase-3/9, BAX and cleaved PARP were upregulated, while Bcl-2 and Bcl-xL were downregulated. All the results indicated that CDCA5 has a protumor effect by inducing the downstream signaling pathway of mitochondrial-mediated apoptosis. Therefore, our data first revealed that the CDCA5-mediated antiapoptotic pathway plays a pivotal role in bladder carcinogenesis.

The PI3K/AKT/mTOR signaling pathway is important for multiple cellular processes, including proliferation, apoptosis, metabolism, migration, invasion and differentiation 39. Phosphatidylinositol 3-kinases (PI3K) are a family of intracellular enzymes capable of phosphorylating the 3'-OH of the inositol ring of phosphatidylinositol (PtdIns). PI3K activates the serine/threonine kinase Akt, which subsequently phosphorylates the mammalian target of rapamycin (mTOR) to regulate cell growth, protein synthesis and apoptosis. Numerous studies have indicated that AKT suppresses apoptosis via the phosphorylation of Bcl2, protease-activated receptor 4 and other proapoptotic proteins 40, 41. In addition, AKT was also reported to play a key role in the progression of BC 42, and the activation of the PI3K/AKT/mTOR signaling pathway contributed to tumor progression and suggested poor clinical prognosis in BC patients 43. Our results demonstrated that CDCA5 knockdown suppressed the PI3K/AKT/mTOR signaling pathway and induced apoptosis and G2/M cell cycle arrest. This partially explains the stunted growth of tumor cells after CDCA5 knockdown. SC79 is a unique and specific AKT activator that induces the phosphorylation of AKT at the Thr308 and Ser473 sites. In our study, SC79 partially rescued the effects of CDCA5 knockdown in BCa cells. SC79 treatment upregulated the expression of p-AKT and p-mTOR in CDCA5-knockdown cells and significantly promoted cell proliferation. These results demonstrated that CDCA5 regulated cellular processes via the PI3K/AKT/mTOR signaling pathway; however, the detailed relationship between CDCA5 and the PI3K/AKT/mTOR pathway and the mechanism of AKT in BC requires further study.

In conclusion, we have identified CDCA5 as a novel functional oncogene in BC, and high CDCA5 expression is associated with an advanced clinical stage and poor prognosis. Our results indicate that CDCA5 regulates apoptosis through the mitochondrial apoptosis pathway and that CDCA5 promotes cell proliferation by regulating cyclins and CDKs; CDCA5-knockdown arrests BC cells in G2/M. All these findings indicated that CDCA5 acts as a tumor promoter in BC. Moreover, CDCA5 may serve as a potential epigenetic biomarker and a novel molecular target for BC.

## Supplementary Material

Supplementary tables.

## Figures and Tables

**Table 1:** Association between the CDCA5 expression level and the clinicopathological features of 408 patients with MIBC from the data in The Cancer Genome Atlas database.

Characteristic	CDCA5 expression levels		P-value
	Low	High	
**Sex**			0.736
Male	149	152	
Female	55	52	
**Age (years)**			1
<65	75	75	
≥65	129	129	
**Tumor grade**			**<0.0001^a※^**
Low	21	2	
High	183	202	
**Stage**			0.092
I or II	75	59	
III or IV	129	145	
**Lymph node metastasis**			0.92
Absent	119	118	
Present	85	86	
**Distant metastasis**			**0.004^b^**
Absent	120	84	
Present	91	120	

^a^P<0.001; ^b^P<0.01. ※ ( Continue Correction); CDCA5, cell division cycle associated 5.
