# Supplementary material for: CDCA5 functions as a tumor promoter in bladder cancer by dysregulating mitochondria-mediated apoptosis, cell cycle regulation and PI3k/AKT/mTOR pathway activation
Source: J Cancer. 2020 Feb 10;11(9):2408–20. doi: 10.7150/jca.35372 (PMC7066009; doi:10.7150/jca.35372)
Supplement: Supplementary file 1 — Supplementary tables. [file jcav11p2408s1.pdf]

Supplementary Table S1: Baseline characteristics of the 20 BC patients

| Patient ID | Sex | Age | BMI | Smoking History | Drinking History | Hematuria | Urine Leucocyte (/μL) | Urine Bacterium (/μL) | Serum creatinine (μmol/L) | Serum urea (mmol/L) | Serum uric acid (μmol/L) |
|---|---|---|---|---|---|---|---|---|---|---|---|
| BC01 | M | 51 | 22.4 | Current | Current | Yes | 755.8 | 15.3 | 105 | 4.9 | 325 |
| BC02 | M | 60 | 21.8 | Never | Never | No | 86.9 | 27.5 | 94 | 6.9 | 356 |
| BC03 | M | 40 | 21.5 | Current | Never | Yes | 21.6 | 386.3 | 126 | 6.4 | 422 |
| BC04 | F | 59 | 20.5 | Never | Never | Yes | 152.7 | 271.4 | 49 | 5.3 | 275 |
| BC05 | M | 78 | 16.0 | Never | Never | Yes | 70.6 | 36.9 | 63 | 4.4 | 209 |
| BC06 | M | 80 | 20.2 | Never | Never | Yes | 60.3 | 26.1 | 318 | 32.5 | 678 |
| BC07 | M | 82 | 24.0 | Never | Current | No | 10.0 | 720.6 | 87 | 4.1 | 300 |
| BC08 | M | 75 | 25.1 | EX | Never | Yes | 154.8 | 1828.7 | 80 | 7.8 | 381 |
| BC09 | M | 77 | 19.9 | Never | Never | No | 86.8 | 14.7 | 98.0 | 8.4 | 123.0 |

| | | | | | | | | | | | |
|---|---|---|---|---|---|---|---|---|---|---|---|
| BC10 | M | 79 | 19.4 | Current | Never | Yes | 1653.9 | 378.8 | 128.0 | 11.0 | 340.0 |
| BC11 | F | 56 | 22.0 | Never | Never | No | 5.8 | 6.6 | 110.0 | 6.6 | 341.0 |
| BC12 | M | 62 | 25.3 | Never | Current | No | 86.5 | 46.5 | 74 | 5.3 | 422 |
| BC13 | F | 65 | 27.0 | Never | Never | Yes | 79.8 | 103.7 | 103 | 9.3 | 525 |
| BC14 | M | 80 | 19.8 | Never | Never | Yes | 1134.4 | 854.7 | 132 | 6.96 | 377 |
| BC15 | M | 69 | 20.0 | Never | Never | Yes | 68.4 | 10.7 | 79 | 2.92 | 102 |
| BC16 | F | 74 | 20.8 | Never | Never | Yes | 1.5 | 24.1 | 85 | 9.31 | 335 |
| BC17 | M | 79 | 26.7 | Never | Never | Yes | 17.7 | 2.6 | 134 | 8.89 | 249 |
| BC18 | M | 60 | 20.8 | Current | Never | No | 39.2 | 9.3 | 70 | 6.19 | 326 |
| BC19 | M | 74 | 21.6 | Current | Current | Yes | 42.3 | 37.5 | 88 | 8.79 | 376 |
| BC20 | M | 58 | 19.5 | Current | Current | Yes | 194.8 | 83.8 | 87 | 8.27 | 264 |

**Supplementary Table S2: Clinicopathologic parameters of the 20 BC patients**

| Patient Id | Recurrence | Multifocality | Invasiveness | Grading | Tumor Size | Cystoscopy Findings |
|---|---|---|---|---|---|---|
| BC01 | No | Yes | Yes | G3 | <2cm | Cauliflower-appearance tumor |
| BC02 | Yes | No | Yes | G1 | ≥2cm | Cauliflower-appearance tumor |
| BC03 | No | No | Yes | G3 | <2cm | Carpet-like swollen lesion |
| BC04 | No | No | Yes | G3 | ≥2cm | Broad base tumor |
| BC05 | No | Yes | Yes | G3 | ≥2cm | Broad base papillary swollen lesion |
| BC06 | No | Yes | Yes | G3 | ≥2cm | Cauliflower-appearance tumor |
| BC07 | Yes | NA | Yes | G2 | NA | - |
| BC08 | Yes | No | Yes | G3 | <2cm | Cauliflower-appearance tumor |
| BC09 | No | NA | Yes | G3 | NA | - |
| BC10 | Yes | NA | Yes | G3 | ≥2cm | Cauliflower-appearance tumor |
| BC11 | No | No | Yes | G3 | NA | - |
| BC12 | Yes | No | Yes | G3 | ≥2cm | Cauliflower-appearance tumor |

| | | | | | | |
|---|---|---|---|---|---|---|
| BC13 | No | No | Yes | G3 | NA | - |
| BC14 | No | No | Yes | G3 | ≥2cm | Broad base cauliflower-appearance tumor |
| BC15 | No | Yes | Yes | G3 | ≥2cm | Cauliflower-appearance tumor |
| BC16 | No | No | Yes | G3 | ≥2cm | Broad base tumor |
| BC17 | Yes | No | Yes | G3 | ≥2cm | Swollen lesion |
| BC18 | No | No | Yes | G3 | ≥2cm | Broad base irregular swollen lesion |
| BC19 | No | Yes | Yes | G3 | ≥2cm | Multiple papillary tumors |
| BC20 | No | No | Yes | G3 | ≥2cm | Tumor like follicular hyperplasia |
